# Single dose oral dexamethasone versus multi-dose prednisolone in the treatment of acute exacerbations of asthma in children who attend the emergency department: study protocol for a randomized controlled trial

**DOI:** 10.1186/1745-6215-13-141

**Published:** 2012-08-21

**Authors:** John Cronin, Una Kennedy, Siobhan McCoy, Sinéad Nic an Fhailí, Gloria Crispino-O’Connell, John Hayden, Abel Wakai, Sean Walsh, Ronan O’Sullivan

**Affiliations:** 1 Paediatric Emergency Research Unit (PERU), Emergency Department, Our Lady’s Children’s Hospital (OLCHC), Crumlin, Dublin 12, Ireland; 2 National Children’s Research Centre, OLCHC, Dublin 12, Ireland; 3 Emergency Department, St James’s Hospital, Dublin 8, Ireland; 4 Emergency Care Research Unit (ECRU), HRB Centre For Primary Care Research, Division of Population Health Sciences (PHS), Royal College of Surgeons in Ireland, 123 St. Stephen’s Green, Dublin 2, Ireland; 5 Department of Paediatrics, University College Dublin (UCD), Belfield, Dublin 4, Ireland

**Keywords:** Asthma, Pediatric, Corticosteroid, Randomized controlled trial, Emergency department

## Abstract

**Background:**

Asthma is a major cause of pediatric morbidity and mortality. In acute exacerbations of asthma, corticosteroids reduce relapses, subsequent hospital admission and the need for ß_2_-agonist therapy. Prednisolone is relatively short-acting with a half-life of 12 to 36 hours, thereby requiring daily dosing. Prolonged treatment course, vomiting and a bitter taste may reduce patient compliance with prednisolone. Dexamethasone is a long-acting corticosteroid with a half-life of 36 to 72 hours. It is used frequently in children with croup and bacterial meningitis, and is well absorbed orally. The purpose of this trial is to examine whether a single dose of oral dexamethasone (0.3 mg/kg) is clinically non-inferior to prednisolone (1 mg/kg/day for three days) in the treatment of exacerbations of asthma in children who attend the Emergency Department.

**Methods/design:**

This is a randomized, non-inferiority, open-label clinical trial. After informed consent with or without assent, patients will be randomized to either oral dexamethasone 0.3 mg/kg stat or prednisolone 1 mg/kg/day for three days. The primary outcome measure is the comparison between the Pediatric Respiratory Assessment Measure (PRAM) across both groups on Day 4. The PRAM score, a validated, responsive and reliable tool to determine asthma severity in children aged 2 to 16 years, will be performed by a clinician blinded to treatment allocation. Secondary outcomes include relapse, hospital admission and requirement for further steroid therapy. Data will be analyzed on an intention-to-treat and a per protocol basis. With a sample size of 232 subjects (105 in each group with an estimated 10% loss to follow-up), we will be able to reject the null hypothesis - that the population means of the experimental and control groups are equal with a probability (power) of 0.9. The Type I error probability associated with this test (of the null hypothesis) is 0.05.

**Discussion:**

This clinical trial may provide evidence that a shorter steroid course using dexamethasone can be used in the treatment of acute pediatric asthma, thus eliminating the issue of compliance to treatment.

**Registration:**

ISRCTN26944158 and EudraCT Number 2010-022001-18

## Background

Asthma is a major cause of pediatric morbidity and mortality. In acute exacerbations of asthma, corticosteroids reduce relapses, subsequent hospital admission and the need for ß_2_-agonist therapy [1]. The earlier corticosteroids are administered in an acute episode, the better the clinical outcome [2]. The British Thoracic Society (BTS) Guidelines on the Management of Asthma (May 2008, revised January 2012) recommend commencing oral prednisolone early for children presenting with exacerbations of asthma and if discharged, continuing treatment for up to three days [3].

Prednisolone is relatively short acting with a half-life of 12 to 36 hours, thereby requiring daily dosing [4]. Outpatient steroid therapy is effective once compliance is assured. However, many factors impact on patient compliance with medication. One study found that at least 7% of children seen in a pediatric emergency department (ED) never have their prescriptions filled [5]. In another study, caregivers of pediatric asthma patients reported adherence to the prescribed length of oral corticosteroid therapy only 64% of the time [6]. Prolonged treatment course, vomiting and a bitter taste may reduce patient compliance with prednisolone [7]. If effective, a single dose of corticosteroid would remove the problem of poor compliance and, therefore, reduce morbidity and the risk of relapse.

Dexamethasone is a long-acting glucocorticoid with a half-life of 36 to 72 hours [4]. It has been used safely in children with croup and bacterial meningitis [8,9], but is not specifically mentioned in the BTS Guidelines [3]. It is well absorbed both orally and parenterally [4]. In a comparative study with a two-day course of hydrocortisone, a single dose of 0.6 mg/kg dexamethasone resulted in a shorter length of hospital stay in children with exacerbations of asthma [10]. Dexamethasone has also been shown to be significantly more palatable than prednisolone to children presenting to the ED with exacerbations of asthma [11]. Whereas intramuscular (IM) dexamethasone is invasive but ensures compliance, a single dose of oral dexamethasone would negate the need for an injection and retain the advantage of ensuring compliance.

Seven randomized controlled trials comparing dexamethasone with prednisolone in the treatment of acute asthma exacerbations in children have been published [12-18]. All of the studies were carried out in the USA, where both the dosing and duration of treatment varies from what is the usual practice in Ireland, the UK and Australasia [19]. The first of these studies compared nebulized dexamethasone with oral prednisolone [18]. Even though no difference was found between the study groups, it is accepted standard practice to give systemic corticosteroids in this clinical setting. Of the other six studies, three compared IM dexamethasone with oral prednisolone [12,13,16]. Again, while the studies found no demonstrable difference between study arms, the utility of an IM injection in a pediatric population with this condition is questionable.

Three studies comparing oral dexamethasone with prednisolone have been published [14,15,17]. Qureshi *et al.* compared ED treatment with an initial dose of oral prednisolone 2 mg/kg (max. 60 mg) followed by 1 mg/kg daily for four days with oral dexamethasone 0.6 mg/kg (max. 16 mg) daily for two days [14]. Altamimi *et al.* compared ED treatment with an initial dose of oral prednisolone 1 mg/kg (max. 30 mg) followed by 1 mg/kg twice daily for five days with a single dose of oral dexamethasone 0.6 mg/kg (max. 18 mg) [15]. Greenberg *et al.* compared ED treatment with a single dose of prednisolone 2 mg/kg (max. 80 mg) followed by 1 mg/kg (max. 30 mg) twice daily for five days with a single dose of 0.6 mg/kg oral dexamethasone (max. 16 mg) followed by one dose of 0.6 mg/kg oral dexamethasone to take the next day and placebo to take twice daily to complete a five-day course [17].

Each of these seven studies outlined found dexamethasone and prednisolone equally effective in treating acute exacerbations of asthma [12-17]. However, there are limitations to each of the trials in terms of study design, sample size and dosing regime, while the age of patients enrolled varied among studies. Asthma is not usually diagnosed before the age of two years because of the prevalence of bronchiolitis in this age group. Ideally all trials included would have studied a homogeneous age group, and included patients up to the age of 16 years. All but one of these studies used a five-day course of prednisolone, whereas the BTS Guidelines recommend a three-day course [3]. Also Chang *et al.* found no difference between a three- and five-day course of prednisolone in children with acute exacerbations of asthma who are not hospitalized [20]. In addition, the lack of a consistent and reliable outcome measure in all of the studies makes the results difficult to interpret. A summary table of these studies can be found in Table 1.

**Table 1:** Studies identified comparing prednisolone with dexamethasone for treatment of acute exacerbations of asthma in children

**Author, date, country**	**Patient group**	**Study type**	**Interventions and control groups**	**Outcomes**	**Follow-up**	**Results**
Scarfone *et al*., 1995, USA [18]	111 children aged one month to seven years with mild-moderate exacerbations of asthma defined by Pulmonary Index score	Single-center, prospective, randomized, double-blind double placebo trial	Oral prednisolone 2 mg/kg (max. 60 mg) for 5 days vs. nebulized dexmethasone 1.5 mg/kg (max. 45 mg) stat	Hospitalization and relapse rates and the rate and degree of clinical improvement, measured by the PI score	Follow-up phone call at 48 hours	111 children completed the study (56 Dex, 55 Pred). 21% of the dexamethasone group required hospitalization, compared to 31% of the prednisolone group. None of the dexamethasone group who were discharged, returned for further treatment compared to 6 of 38 (16%) prednisolone-treated patients.
Klig *et al*., 1997, USA [12]	44 children aged 3 to 16 years with more than two previous episodes of wheezing and mild to moderate wheezing episodes defined by pulmonary indexscore 2 to 7 and SaO_2_ ≥95%	Single-center, prospective, randomized pilot study	ED treatment with oral prednisolone 2 mg/kg (max. 100 mg) followed by 1mg/kg x two days vs. single dose of IM dexamethasone 0.3 mg/kg (max. 15 mg)	Primary outcome symptomatic improvement and relapse or deterioration defined by patients and parents; secondaryoutcomes PIS on ED discharge and further corticosteroid use after follow-up	Follow-up in clinic or by telephone on Day 5 after discharge from ED by physician blinded to patient allocation	42 children participated in the study. No significant differences between Dex and Pred groups were found for gender or age. Clinical progress (based on PI) with treatment in ED was the same in both groups: pre-treatment median, PI = 6; ED discharge median, PI = 2. None of study patients hospitalzsed during the follow-up period, and all reported symptomatic improvement since initial treatment.
Gries *et al.,* 2000, USA [13]	33 children aged six months to seven years with mild to moderate exacerbations of asthma defined by clinicalasthma score	Single-center, prospective, randomized investigator-blinded trial	Oral prednisolone 1 mg/kg (max. 20 mg) bd x 5 days or single dose of IM dexamethasone acetate 1.7 mg/kg (max. 36 mg)	Primary outcome change in clinical asthma score, improvement in clinical status, use of bronchodilator and tolerance of steroid; secondary outcomes subjective clinical asthma score, personality changeand tolerance of medication	Clinic visits and telephone contacts on Days 3 and 7 to review parents’ diary entries. Clinic visits on Days 5 and 14, history of symptoms, diary entries and examination. Telephone contact atDay 28 to assess relapse and satisfaction. Day 14 urine cortisol-to- creatinine ratio	15 patients in the Dex group and 17 in the Pred group completed the study. Clinical asthma score improved significantly in both groups during the first five days of therapy, and no significant difference was seen in the rate of improvement between the two groups. Three patients refused more than 75% of pred doses, and another four missed 30% to 50% of doses despite parents’ best efforts. IM caused no complications, and approx. 70% of parents in both groups stated that they would choose IM Dex to treat the next asthma exacerbation.
Qureshi *et al.,* 2001, USA [14]	628 patients aged 2 to 18 years with a history of asthma presenting with exacerbation, defined as worsening symptoms or increased difficulty breathing with worsening PEFR	Single-center, prospective randomized trial	ED treatment with single dose of oral prednisolone 2 mg/kg (max. 60 mg) followed by 1 mg/kg od for four days vs. ora l dexamethasone 0.6 mg/kg(max 16 mg) od x 2 days	Primary outcome wasrelapse rate; secondary outcomes: hospitalization, vomiting, medication compliance, persistence of symptoms, school or work days missed	A research assistant contacted each patient’s family at 11 to 14 days after ED discharge to obtain follow-up data	When Dex compared with Pred, relapse rates (7.4% of 272 vs. 6.9% of 261), hospitalization rates from ED (11% vs. 12%) or after relapse (20% vs. 17%), and symptom persistence at 10 days (22% vs. 21%) were similar. In the Pred group more children were excluded for vomiting in ED (3% vs. 0.3%; *P* = .008), more parents were noncompliant (4% vs. 0.4%; *P* = .004), and more children missed ≥2 days of school (19.5% vs. 13.2%; *P* = .05).
Altamimi *et al.,* 2006, USA [15]	117 patients aged 2 to 16 years with mild to moderate exacerbation of asthma, defined as pulmonary index score <9 or PEFR >60%	Single-center prospective randomized double-blind trial	ED treatment with single dose of oral prednisolone 1 mg/kg (max. 30 mg) followed by 1 mg/kg bd for 5 days vs. oral dexamethasone 0.6 mg/kg (max. 18 mg) x single dose only	Primary outcome was the number of days for PSAS to return to baseline or PEFR to return to >80% predicted; secondary outcomes were short-term improvement in PEFR and PIS at discharge from ED, time to discharge, number of salbutamol therapies in ED and at home, unexpected returns to ED, need for admission after discharge and improvement in PIS on Day 5	Telephone call at 48 hrs to assess progress using standard assessment form. Re-assessment at Day 5 of salbutamol treatments, side effects, unscheduled visits to a doctor, review of PSAS sheet, examination and compliance. Ifsymptoms were ongoing at Day 5, patient had weeklyphone call for three weeks	Baseline characteristics similar. Mean no. days needed for PSAS return to baseline (0 to 0.5) in the Dex and Pred groups were 5.21vs 5.22 days, respectively (mean difference, -0.01; CI, -0.70, 0.68). PI scores similar in both groups at initial presentation, initial ED discharge and at Day 5 follow-up visit. At first visit, mean time to discharge was 3.5 h (± 1.93) for Dex and 4.3 h (± 3.67) for Pred (mean difference, -0.8; CI, -1.8, 0.2). Initial admission rate 9% (Dex) versus 13.4% (Pred). No significant difference in no. salbutamol therapies needed in ED or at home after discharge. For subjects discharged home, admission rate after initial discharge was 4.9% (Dex) vs. 1.8% (Pred), resulting in overall hospital admission rates of 13.4% (Dex) and 14.9% (Pred).
Gordon *et al.,* 2007, USA [16]	194 children aged 18 months to 7 years with moderate exacerbations of asthma defined as clinical asthma score 3 to 7	Single-center, prospective, randomized trial	Oral prednisolone 2 mg/kg (max. 50 mg) daily x 5 days vs. single dose of IM dexamethasone 0.6 mg/kg (max. 15 mg) IM	Primary outcome waschange in asthma scorefrom presentation to score at four-day follow-up. Secondary outcomes were hospital admission on first presentation, by four days and by two weeks, respiratory symptoms at four days and need for unplanned medical visits during two weeks post enrollment	Scheduled follow-up at 96 to 120 hrs post-enrollment andtelephone interview at two weeks to assess secondary outcomes	89 patients randomized to dex and 93 to pred. Group characteristics similar at baseline. Among those discharged from the ED, 62 (90%) of 69 and 64 (90%) of 74 patients in the dex and pred groups, respectively, were reassessed at four days for primary outcome. Mean change in total asthma score 3.6 in dex group and 3.4 in pred group (difference, 0.2; 95% CI, -0.4 to 0.7). Of pts initially discharged, 5.9% of dex pts and 4.1% of pred pts were admitted before two-week follow-up (difference, 1.8%; 95% CI, -5.4% to 9.0%).
Greenberg *et al*., 2008, USA [17]	89 children aged 2 to 18 years with any exacerbation of asthma	Prospective, randomized, double-blind conveniencestudy at urban, tertiary care, academic children’s hospital ED	0.6 mg/kg of oral dexamethasone (max dose 16 mg) or 2 mg/kg of oral prednisone (max. 80 mg) during ED treatment. At ED discharge, pts in dex arm received one dose of 0.6 mg/kg dex to take next day and placebo to take BD tocomplete five-day course.Pts in pred arm received 1 mg/kg pred to take twice daily for five days.	Primary outcome wasrelapse within 10 days, defined as need for subsequent hospitalizationor unscheduled visit with a medical provider. Secondary outcome was emesis with steroid administration in the ED.	Patients and their families were called by telephone 10 days after their ED visit for follow-up.	89 patients completed study: 38 in pred group and 51 in dex group. 3 patients in pred group (8%) and 8 patients in dex group (16%) required an unscheduled follow-up visit (*P* = .27). 7 patients in pred group (18%) and 5 patients in dex group (10%) had vomiting (*P* = .24). No difference found in relapse rate or incidence of vomiting between patients given pred and dex for asthma exacerbations.

There are a number of clinical asthma scores which have been employed as outcome measures in pediatric asthma research. Of these, the Pediatric Respiratory Assessment Measure (PRAM) [21], a validated, responsive and reliable tool to determine asthma severity in children aged 2 to 16 years (Table 2) [22], appears to be the most appropriate as an application in the emergency care setting [23].

**Table 2:** The Pediatric Respiratory Assessment Measure (PRAM)

**Signs**	**0**	**1**	**2**	**3**
Suprasternal muscle contraction	Absent		Present	
Scalene muscle contraction	Absent		Present	
Air entry*	Normal	Decreased at bases	Widespread decrease	Absent/minimal
Wheezing*	Absent	Expiratory only	Inspiratory and expiratory	Audible without stethoscope/silent chest with minimal air entry
O_2_ saturation	≥95%	92%-94%	<92%	

*in case of asymmetry, the worst lung is rated.

The PRAM score consists of five components and has a maximum total of 12 points: suprasternal retractions (0 to 2), scalene muscle contraction (0 to 2), air entry (0 to 3), wheezing (0 to 3) and O_2_ saturation (0 to 2).

### Study aims

The hypothesis to be tested is whether a single dose of oral dexamethasone 0.3 mg/kg (max. 12 mg) is non-inferior to prednisolone 1 mg/kg/day (max. 40 mg) for three days in the treatment of exacerbations of asthma in children, as measured by the Pediatric Respiratory Assessment Measure (PRAM). This dosing regime is more reflective of current prescribing practices in pediatric emergency medicine in the UK and Ireland and also in Australasia [19].

## Methods/design

### Study design and setting

This is a randomized, open-label, active control clinical trial. It will be conducted at a single tertiary urban pediatric ED (Our Lady’s Children’s Hospital Crumlin (OLCHC)) in Dublin, Ireland.

## Ethical considerations

This study will be performed in accordance with Good Clinical Practice (GCP) Guidelines, the EU CT Directive 2001/20/EC, GCP Commission Directive 2005/28/EC, the Declaration of Helsinki (2008) and with all other local regulatory requirements. Risk analysis was carried out as part of the protocol development. The study protocol was approved by the Health Research Ethics Committee (HREC) at OLCHC, Dublin.

### Regulatory considerations

The Irish Medicines Board (IMB) is the competent authority for the review and approval of clinical trials with an investigational medicinal product in Ireland. The trial was approved by the IMB on the 3 June 2011. The trial’s EudraCT Number is 2010-022001-18. The trial has been registered with http://www.controlled-trials.com (registration number is ISRCTN26944158).

### Subject selection

The eligible participants will be all children aged 2 to 16 years who present to the ED of OLCHC, Dublin 12, Ireland with an exacerbation of asthma (as defined below) and who fulfill the inclusion and exclusion criteria below.

### Inclusion and exclusion criteria

Inclusion and exclusion criteria are presented below (Table 3).

**Table 3:** Inclusion and exclusion criteria

***Inclusion criteria*:**	**· Ages 2 to 16 years**
	**· Background history of asthma (as defined below)**
	**· Presentation with an asthma exacerbation (as defined below)**
*Exclusion Criteria:*	· Less than 2 years old or over 16 years
	· Critical or life-threatening asthma (as defined below)
	· Known TB exposure
	· Active varicella or herpes simplex infection
	· Documented concurrent infection with RSV
	· Fever >39.5°C
	· Use of oral corticosteroids in the previous four weeks
	· Concurrent stridor
	· Galactose intolerance, the Lapp-lactase deficiency or glucose-galactose malabsorption
	· Significant co-morbid disease: lung, cardiac, immune, liver, endocrine, neurological or psychiatric

### Definition of an asthma exacerbation

An exacerbation of asthma, for the purpose of this study, will be defined as acute asthma, which prompts assessment at the ED, and has any, or all, of the following clinical features:

Dyspnea

Wheeze

Acute cough

Increased work of breathing

Increased requirement for ß_2_-agonist from baseline use

O_2_ saturation <95%

### Definition of a “background history of asthma”

Asthma for the purpose of this study will be defined as either:

at least one previous episode of ß_2_-agonist-responsive wheeze in a child two years of age or over; or

a prior diagnosis of asthma, made by a pediatrician, or clinician of comparable experience.

### Definition of a critical or life-threatening asthma exacerbation

For the purpose of this study, critical or life-threatening asthma will be defined as per the OLCHC ED Asthma Guideline, that is, patients displaying one or more of the following clinical features:

Confused/drowsy

Maximal accessory muscle use/recession

Poor respiratory effort (including bradypnea)

Exhaustion

Silent chest

Cyanosis

O_2_ saturation < 90% in air

Marked tachycardia

Unable to verbalize normally (that is, different from baseline verbal ability)

Pneumothorax

### Study medication

#### Form

Prednesol® 5 mg tablets (Phoenix Labs Ltd., Cahill May Roberts, Pharmapark, Chapelizod, Dublin 20) will be used for the prednisolone group. They are pink, circular, flat, bevel-edged scored tablets, and each tablet contains 5 mg of prednisolone as prednisolone sodium phosphate. Study patients can swallow the Prednesol® tablets whole, but they will be dissolved in water for younger patients.

Dexamethasone tablets 2 mg (Organon Ireland Ltd., Drynam Road, Swords, Co. Dublin) will be used for the dexamethasone group. Each tablet contains 2 mg of dexamethasone. Each tablet is a round, 6 mm, flat, white tablet with the code “XC/8” engraved on one surface and “Organon*” on the other. Again, these tablets can be swallowed whole, but they will be crushed and dispersed in water for younger patients.

#### Dosing schedule

The dose of prednisolone will be rounded off to the nearest 5 mg (for example, a child weighing 23 kg will receive 25 mg, and a child weighing 21 kg will receive 20 mg of prednisolone). The dose of dexamethasone will be rounded off to the nearest 2 mg. This is detailed in Table 4. This process of ‘rounding off’ is routine clinical practice in our ED when prescribing oral corticosteroids, for example, prednisolone in asthma, dexamethasone in croup.

**Table 4:** Dosing schedule

**Prednisolone dosing schedule**	
**Weight (kg)**	**Prednisolone (mg)**
8 to 12	10
13 to 17	15
18 to 22	20
23 to 27	25
28 to 32	30
33 to 37	35
≥38	40
**Dexamethasone dosing schedule**	
≤9	2
10 to 16	4
17 to 23	6
24 to 29	8
30 to 36	10
≥37	12

#### Investigational medicinal product storage and handling procedures

The principal investigator (PI) or designee will be responsible for maintaining and documenting records for receipt, dispensing, accountability, collection and destruction of all investigational medicinal products (IMP). The PI or designee shall only use the IMP provided for the participants involved in the study.

Study packs will be made up by the Pharmacy Department at OLCHC. Packs will be made up for each medication according to weight band, that is, for prednisolone there will be 10, 15, 20, 25, 30, 35 and 40 mg packs and for dexamethasone there will be 2, 4, 6, 8, 10 and 12 mg packs. The pack will be made up of a small white cardboard box containing the medications. Each prednisolone pack will contain three doses of the medication. Prednisolone tablets will be dispensed in their original foil blisters. The foil blisters making up each dose will be dispensed in smaller clear plastic zip-lock bags within the outer bag. Each of these smaller bags will be labeled appropriately. Each dexamethasone pack will contain one dose in a screw cap and labeled tablet vial. Each study pack will be clearly labeled with the title of the study, the name of the medication (“PREDNISOLONE” or “DEXAMETHASONE”), the dose contained (for example, “10 mg”) including the number of doses, and the medication batch number.

The study packs shall be stored in a secure area with restricted access. A supply of each type of study pack (as described above) will be maintained in a locked cupboard (with restricted access) in the ED, in order to ensure immediate availability when a patient is enrolled into the study.

The site will maintain records of the storage temperature to ensure compliance with the study specified ranges. A copy of the temperature records will be included in the study files. If the site personnel observe a deviation from the specified temperature, this shall be reported to the sponsor.

The study monitor will review IMP storage and handling procedures on a regular basis at site monitoring visits.

#### Identity and labeling of investigational product(s)

Prednesol® 5 mg tablets and Dexamethasone tablets 2 mg will be supplied as open label commercial stock and will be labeled and stored in accordance with the required regulatory guidelines, the Summary of Product Characteristics and hospital procedures.

#### Storage of second and third doses of prednisolone

After randomization and administration of the Day 1 dose, and prior to discharge from the ED, the parents/legal guardians of patients randomized to the prednisolone group will be instructed on how to store the remaining study drug appropriately and out of reach of children. If a patient randomized to the prednisolone group is admitted to OLCHC, a member of the research team will be responsible for supplying the prednisolone dose on Days 2 and 3. This dose will be dispensed from the study medication pack.

### Study procedure

#### General description

Before the start of the study, the research team will conduct training sessions for all ED nurses and physicians to explain roles and responsibilities and all aspects of the study. Training sessions on the PRAM score have also taken place, and this score has been used in the assessment of all asthma exacerbations (over the age of 2) presenting to the ED from April 2011 onwards.

Eligible participants will be identified at triage or during clinical consultation by the ED clinician. They and their parents/guardians will be provided with written and verbal information about the study. All patients in the study will be recruited by a member of the research team or an ED clinician.

After eligibility for inclusion in the study is confirmed, the research trial and procedures involved will also be explained to the patient in age-appropriate language. In all cases, where appropriate, informed assent will be obtained from the patient. This may not be possible in younger patients and in patients who are too unwell (for example, short of breath) to actively participate in the consent process. If the patient recovers sufficiently, we will go through the assent process with them at a later stage. In all cases without exception, informed consent will be obtained from the parent/legal guardian. If a patient refuses assent they will not be included in the study, even if a parent/legal guardian gives consent.

The informed consent process will be documented in the medical notes (including the date/time of consent and the provision of information to the patient/parent(s)/guardian). Once the patient is consented, randomization will occur.

The patient will be randomized by the recruiting clinician to receive either a stat dose of oral dexamethasone 0.3 mg/kg (max. dose 12 mg) in the ED, *or* prednisolone 1 mg/kg/day (max. dose 40 mg) for three days. The clinician will pick the next available card from a pre-randomized pack of study cards contained in a locked storage cupboard in the ED. This card will contain the study number (that is, Subject ID) of that particular patient and will state to which treatment arm they are assigned (“DEXAMETHASONE” or “PREDNISOLONE”). Subject IDs will be allocated on a consecutive basis of recruitment to the study. The patient’s weight will be checked (all patients presenting to OLCHC ED are routinely weighed after arrival unless in extremis) and the appropriate dose of study medication will then be read from the Dosing Schedule (see Table 4). The next pack of study medication with that dose will be taken. The first dose will be given in the ED by the member of the recruiting clinician. If the patient is assigned to the dexamethasone group, then this is the only dose that will be given. If the patient is assigned to the prednisolone group, the second and third doses (each dose in original foil blistered strip contained in separate labeled clear plastic zip-lock bags as described) will be given to the patient and their parent/guardian prior to discharge.

If the patient vomits the medication dose given in ED within 30 minutes of administration, a second dose will be supplied. If the patient vomits again within 30 minutes of the second dose, then no further dose will be given but the patient will remain in the study and be analyzed on an intention-to-treat basis.

Subjects and parents/legal guardians will be trained on the correct administration of prednisolone on Days 2 and 3. This training will be documented in the subject’s medical notes. Patients will be requested to bring the study packs with them for the Day 4 assessment.

### Patient randomization

The randomization process has been designed by CSTAR (Health Research Board Centre for Support and Training in Analysis and Research). Randomization will occur in blocks of 12 subjects. An equal number of subjects will be randomized to each treatment group for every 12 subjects enrolled.

The clinician will pick the next available envelope from a pre-randomized pack of study envelopes contained in a locked storage cupboard in the ED. This envelope will contain the study number (that is, Subject ID) of that particular patient and will contain a card which states to which treatment arm they are assigned (“DEXAMETHASONE” or “PREDNISOLONE”). Subject IDs will be allocated on a consecutive basis of recruitment to the study.

### Outcome measures

The outcome measures used to compare differences between the study arms will include:

Primary

PRAM score at Day 4

Secondary

Relapse rate – a relapse will be defined as any visit to a healthcare provider, for example, General Practitioner, ED, as a result of asthma symptoms within 14 days of study enrolment

Number of salbutamol therapies given following enrolment

Side effects, in particular, vomiting

Compliance with medication, assessed by interview and the return of study packs

### Statistical considerations

The aim of this study is to demonstrate that a single dose of dexamethasone is clinically non-inferior to a three-day course of prednisolone.

### Sample size and power

In this study, the Primary Variable is the PRAM Score at discharge and at Day 4 after discharge. It is assumed that the distribution of PRAM scores will take an approximate normal distribution. A change in PRAM score from 6.57 ± 1.5 (mean ± standard deviation) at triage to 2.93 ± 2.23 at discharge from ED was the basis of the validation study on PRAM (personal communication from Francine Ducharme) [21]. The sample size calculation below compares dexamethasone and prednisolone at Day 4. It assumes that dexamethasone is not inferior to prednisolone if the average PRAM score for dexamethasone at Day 4 is not more than one point higher than the prednisolone average PRAM score. On this basis, with a sample size of 232 subjects (105 in each group with a 10% loss to follow-up) we will be able to reject the null hypothesis - that the population means of the experimental and control groups are equal with a probability (power) of 0.9. The Type I error probability associated with this test (of the null hypothesis) is 0.05.

### Data analysis

Data will be analyzed on an intention-to-treat and a per protocol basis. Data will be presented as means with standard deviations and Student’s *t* test will be used to compare differences in proportions between the groups. Significance will be set at the 5% level. No interim evaluation of the data is planned.

### Bias and confounding variables

In terms of selection bias, we feel that this study targets a representative patient population to whom this research ultimately may be clinically applicable. Every effort will be made to ensure that recruitment of participants occurs over all 24-hour periods and at weekends by having patients recruited by the ED physician treating the patient.

We anticipate that the randomized controlled design of this study will minimize the effect of confounding variables on our analysis. However, all potential confounders (that is, age, asthma severity, concomitant medications) will be measured during data collection and they will be adjusted for during the analysis should there be any imbalance. Also, every effort will be made to ensure that the physician who is assessing the primary outcome is blinded to the treatment allocation.

### Safety reporting

All adverse events (AEs) that occur during the study period observed by one of the clinical staff, or reported by the patient or parent/guardian spontaneously, or in response to a direct question, will be noted on the appropriate form. These forms are de-identified. The following procedures will take place depending on the type of event that has occurred.

### Adverse event (AE)

Each AE will be recorded by a member of the research team on an AE Form. AEs will be classified on the form in terms of their severity, association with the study drug, expectedness and seriousness. They will be recorded on an AE Log. The AEs will be reported to the Sponsor and the institutional HREC on a yearly basis as part of an annual safety report and at the end of the trial.

### Serious adverse event (SAE)

Each SAE will be recorded by a member of the research team on an SAE Form. SAEs will be classified on the form in terms of their severity, relatedness to the study drug and expectedness. They will be recorded on a SAE Log. All SAEs will be reported on the SAE form within 24 hours to the Sponsor and the Research Ethics Committee. The research team will ensure that follow-up information and a detailed written report are provided when available.

### Suspected unexpected serious adverse drug reaction (SUSAR)

Each SUSAR will also require expedited reporting to the Sponsor. This will occur as soon as possible, but no later than 24 hours after a member of the research team has first knowledge of the minimum criteria for expedited reporting. In each case, relevant follow-up information should be sought and a detailed, written report completed as soon as possible. It is the Sponsor’s responsibility to ensure that all relevant and available information is forwarded to the Competent Authority (Irish Medicines Board) and the appropriate health ethics committee (HREC, OLCHC). For fatal or life-threatening events this will be done as soon as possible and not later than seven days after the Sponsor becomes aware of the event. Additional relevant information will be sent within eight days of the first report. This will be sent no later than an additional 15 calendar days. For AEs that are not fatal or life-threatening, the Sponsor will ensure that a SUSAR is reported as soon as possible and in any event not later than 15 days after the Sponsor is first aware of the event.

The parents of participants will be provided with 24-hour contact details of a study representative if they have concerns about any component of the study or their child’s condition. We will also report to the hospital Risk Management Team and the Drugs Advisory Committee of the study site.

### Trial Status

Recruitment for the trial completed in July 2012. Data management procedures are underway and statistical analysis will commence in September 2012.

## Abbreviations

AE: Adverse event; BTS: British Thoracic Society; ED: Emergency department; GCP: Good clinical practice; HREC: Health Research Ethics Committee; IM: Intramuscular; IMB: Irish Medicines Board; IMP: Investigational medicinal products; OLCHC: Our Lady’s Children’s Hospital, Crumlin; PI: Principal investigator; PRAM: Pediatric respiratory assessment measure; SAE: Serious adverse event; SUSAR: Suspected unexpected serious adverse drug reaction.

## Competing interests

The authors declare that they have no competing interests.

## Authors’ contributions

ROS conceived the study. JC, UK, SMC, JH, AW, SW and ROS each made substantial contributions to study design; have been involved in drafting the manuscript and revising it critically for intellectual content; and have given final approval of the version to be published. JH provided pharmaceutical support and was involved in creating the study medication packs, GC-OC provided the statistical support and contributed to the drafting of the manuscript. This forms part of JC’s PhD thesis registered with University College Dublin, Ireland. All authors read and approved the final manuscript.
